# High Value-Added Products from Food Waste

**DOI:** 10.3390/foods12214038

**Published:** 2023-11-06

**Authors:** Paula Toshimi Matumoto-Pintro, Bianka Rocha Saraiva

**Affiliations:** 1 Agronomy Department, Graduate Food Science Program and Graduate Animal Science Program, State University of Maringá, Maringá 87020-900, Paraná, Brazil; 2 Graduate Animal Science Program, State University of Maringá, Maringá 87020-900, Paraná, Brazil; bianka_saraiva@hotmail.com

## 1. Introduction

Knowledge and use of technologies can transform waste into sustainable solutions. Among the main types of waste that can be reused are agricultural or crop waste, and agro-industrial waste, largely composed of leaves, stems, peels, pulp, seeds, peels, and bagasse, can be reused due to their nutritional composition and the presence of phytochemical compounds they still contain. They can be used for composting and animal feed, but when they are not reused, they end up being discarded. Proper disposal of this waste requires prior treatment, as it is necessary to reduce its biological load to avoid possible environmental contamination when disposing of it in water bodies; however, this procedure requires greater time and financial resources, which leads to waste being incorrectly disposed of, buried in the ground, or burned, which also contributes to environmental impacts [1].

The reuse of waste, precisely due to the composition of nutrients and compounds they possess, can add value and contribute to the subsistence of farmers and increased incomes in agro-industries as a sustainable alternative for small, medium, and large farms and food industries. The use of waste is also a sustainable way to increase food availability without increasing the agriculture production area and energy expenditure on production factors (such as seedlings, water, tractors, employees, equipment, electrical energy for production, etc.). The estimated global population increase between 2012 and 2050 is approximately 14%, while the estimated increase for 2100 is approximately 40% [2]. These numbers have raised concerns about food security [3] and the sustainability of food production. To meet these demand, it will be necessary to optimize the existing production processes and increase food production. However, natural resources and physical spaces capable of cultivation have limitations, and increased production leads to greater waste generation, which are often solid, humid, and bulky waste.

The characterization of waste, that is, the study and evaluation of its properties, is fundamental for making decisions regarding the types of technologies that can be explored to add value to the material; knowing its chemical composition, its technological properties, the presence of bioactive compounds and antioxidant activity, among other properties of interest, can awaken new directions for the residue, which will then be used as a raw material.

The search for new sources of bioactive compounds and antioxidants [4,5], proteins and essential amino acids [6,7], and bioactive peptides [8] from waste has been increasing, whether for use in isolation or in enrichment or development of innovative food products. When inserting new ingredients into a food matrix, several technological and nutritional interactions/changes can occur, such as protein, amino acid, and carbohydrate availability, and polyphenol–protein, carbohydrate–protein, and protein—lipid interactions, among others. These phenomena can be evaluated during the development of new ingredients, additives, and products, to explain their properties and expected behaviors during use in product development or during storage, preparation, and consumption. The development of technologies to produce value-added products from waste meets the principles of the circular economy and will contribute to increasing the supply of ingredients, additives, and/or products in a sustainable and safe way for industry and farm producers.

## 2. Sustainability

Research seeks to be increasingly aligned with the United Nations (UN) Sustainable Development Goals (SDGs) to develop methodologies that promote sustainability and technological innovation for food production. Sustainability must be present at all stages of production chains, for example, using waste from the production process or waste that is lost during production. The reuse of waste in food production and the development of new products can be further explored. With the use of new technologies, new properties can be characterized and/or improved. Food and nutritional security and the population’s quality of life can be improved through the development of functional food ingredients and additives from waste, which can diversify the food supply.

## 3. Ingredients and Additives

The industry has been increasingly seeking natural ingredients to preserve, enrich, and color foods, and from sustainable sources, characteristics which are requested by consumers who are aware of the issues of the impact they have on the planet and the search for healthy food choices (to benefit health and well-being). Waste is an alternative and potential material for developing new food ingredients.

By-products or waste have a composition rich in nutrients, bioactive compounds, and antioxidant activity, which, with the use of the appropriate technologies and processing, can be reused as alternative ingredients in the food industry. These ingredients can act as a source of proteins or as ingredients and additives for the production or improvement of different types of food. Increasing the supply of additives or natural ingredients can help develop healthier and more sustainable foods. Different types of waste have been used to demonstrate the potential that waste has as a source of sustainable ingredients [9,10,11,12].

This Special Issue includes articles on new ingredients and products with high added value that originate from food waste, whether agricultural or agro-industrial, and their role in food security and sustainability.
